# On the determinants of journal rejection rates: evidence from the Journal of Financial Economics

**DOI:** 10.1186/s40854-026-00908-x

**Published:** 2026-02-03

**Authors:** Karel Hrazdil, Pavel Král, Jiri Novak, Nattavut Suwanyangyuan

**Affiliations:** 1 Beedie School of Business, Simon Fraser University, 8888 University Drive, Burnaby, BC V5A1S6 Canada; 2 Prague University of Economics and Business, Nám. Winstona Churchilla 1938/4, 13067 Prague, Czech Republic; 3 Faculty of Social Sciences, Institute of Economic Studies, Charles University, Opletalova 26, 11000 Prague, Czech Republic; 4 Goodman School of Business, Brock University, 1812 Sir Isaac Brock Way, St. Catharines, ON L2S3A1 Canada

**Keywords:** Rejection rates, Review process, Journal of Financial Economics, A14, I23

## Abstract

We examine how academic journal reviewers’ experience with the peer-review process influences their propensity to recommend manuscript acceptance or rejection. We use data on the total recommended rejections and acceptances for all referees who reviewed at least one paper for the *Journal of Financial Economics (JFE)* between 1994 and 2020. We show that reviewers who write more reports are more likely to recommend the acceptance of manuscripts. We also find that older reviewers, those who graduated from or are affiliated with prestigious universities, and those with more and highly cited publications are more likely to recommend acceptance. There is also some evidence that reviewers with doctoral training in economics, mathematics, physics, and engineering are more likely to recommend acceptance than those with a PhD in finance. We find no consistent evidence of significant differences between genders or among reviewer demographic characteristics. We also document that reviewers who themselves publish more successfully in JFE and publish highly cited articles are, *ceteris paribus*, more likely to recommend rejection of reviewed manuscripts. Our study utilizes a unique research setting to gain new insights into the determinants of the peer-review process in scientific journals.

## Introduction

The culture of academic research is often dominated by the pressure of the frequently repeated “publish or perish” mantra. Peer review is the most widely accepted method for evaluating scientific works, extending beyond publication in academic journals to influence grant allocations, academic promotions, and career trajectories (Jefferson et al. 2002; Shopovski et al. 2020). Those who review papers submitted to academic journals—although typically anonymous—play a crucial role in this process, essentially determining the success or failure of researchers within their disciplines. Despite its longstanding tradition and the pivotal role of reviewers, the peer-review system has notable flaws (Smith 2006; Tsang 2013; Carter 2017; Allen et al. 2022; Aczel et al. 2025), and several studies have identified referee characteristics, biases, and conflicts of interest that negatively impact the outcome of the review process (Kliewer et al. 2005; Brogaard et al. 2014; Hug 2024).

Given that publications are a key determinant of scientific development and academic career progression, the literature on the academic review process itself is vast. Previous studies have investigated various aspects of the review process for economic journals, including author characteristics (Blank 1991), editorial referee screening behaviors (Laband 1990), submission-to-acceptance times (Hamermesh 1994), and author diversity (Cotton 2013). More recent studies have explored shifts in coauthorship trends (Hamermesh 2013), the influence of modifications to the publication process on research productivity (Baghestanian and Popov 2018), and how publishing peer-review reports can affect referee behavior (Bravo et al. 2019). While many journals disclose acceptance rates and submission timelines, pertinent secondary data, such as referee identities, selection processes, and review durations, are rarely made publicly available (Baghestanian and Popov 2018).

This study analyzes whether referees’ recommendations are impacted by their previous experience with publishing and reviewing for a journal. We exploit a large dataset comprising the outcomes of 23,929 reviews conducted by 1,941 individual referees between 1994 and 2020 for the *Journal of Financial Economics* (*JFE*), recognized alongside the *Journal of Finance (JF)* and the *Review of Financial Studies* as one of the most prominent academic journals in the field. The dataset, available in the online data appendix (Schwert 2021b), accompanies Schwert’s (2021a) analysis of shifts in the demand for and supply of academic research in finance over the past several decades. Schwert extensively examines various aspects of papers published in the *JFE*, highlighting notable changes in submission fees, turnaround times, paper length, research themes, and author demographics over time, including factors that influence desk rejection decisions, final acceptance determinations, and study citations. However, Schwert does not examine the factors that influence a referee’s inclination to recommend manuscript acceptance or rejection. Despite not providing detailed data on the characteristics of submissions, referee report completion years, and referees’ own publication records in other journals, this unique aggregate dataset still enables us to show that the likelihood that a paper will be recommended for acceptance by a referee rises significantly with the number of referee reports.

These results provide new evidence and make important contributions to the literature on the determinants of referee recommendation decisions (Laband 1990; Schultz 2010; Welch 2014; Schwert 2021a). Our findings are consistent with the socioemotional selectivity theory proposed by Carstensen et al. (1999), which proposes that as people grow older, they become more selective in their social relationships, prioritizing emotionally meaningful interactions, and the interdependence theory, summarized by Van Lange and Balliet (2014), which posits that individuals weigh the costs and benefits of cooperation versus competition in their interactions with others. Both theories suggest that more experienced and well-established referees may be less focused on competition and more interested in collaboration and helping others succeed.

## Prior literature

Academics occupy two roles during the peer-review publication process: one as authors who produce research and the other as editors and referees who evaluate the quality of research produced by their peers (Lawrence 2003). While some studies explore how editors can improve the quality of a journal’s pool of submitted manuscripts (Baghestanian and Popov 2018; Edwards and Leigh 2022) and analyze referees’ motives for participating in the review process (Engers and Gans 1998; Lee and Brudney 2009), others focus on identifying the various factors that influence referees’ recommendations. For example, prior studies show that acceptance rates can be influenced by increasing expectations for methodological rigor and editors’ aspirations for the journal to have a greater impact (Edwards and Leigh 2022), the number of referees and their level of agreement—with more reviewer agreement potentially leading to higher acceptance rates (Schultz 2010; Welch 2014)—the editorial process, which dictates publication speed, referees’ burdens, quality control (Kovanis et al. 2016), and a reviewer’s publication history, although the volume of reviews provided each year may not necessarily differ based on whether or not a referee has published in top-tier journals (Aarssen et al. 2009).

All submissions to the *JFE* undergo an initial editorial review to assess their suitability for publication in the journal. If deemed appropriate and not ‘desk rejected’ (i.e., unlikely to become publishable in the *JFE* [Schwert 2021a]), the manuscript is then typically forwarded to at least one independent reviewer for expert evaluation of its scientific quality (Elsevier 2024). The journal peer-review process is largely standardized, with referees used by the *JFE* being carefully chosen from the pool of qualified experts in the field; however, these experts differ in terms of their experience refereeing for and publishing in the *JFE*.

The association between prior experience with publishing and refereeing for the *JFE* and the propensity to accept a manuscript under review for the journal is not a priori obvious (Schultz 2010; Welch 2014; Edwards and Leigh 2022; Hamermesh 2013). On the one hand, more experienced reviewers may be more knowledgeable about methodological rigor and the relevant research literature and therefore may be more skeptical about a new manuscript’s original contribution or empirical results, potentially increasing the likelihood of recommending rejection. However, they may also be able to better evaluate the strengths and weaknesses of manuscripts under review and guide the authors toward eventual paper acceptance by making constructive suggestions. On the other hand, less experienced referees may feel pressured to demonstrate their competence by being more critical, might be less confident and more cautious in their judgment, and more prone to errors in assessing a manuscript’s quality, leading them to reject submissions that could be improved with revisions. Alternatively, less experienced referees might approach manuscripts with a more optimistic perspective, apply less stringent criteria or have lower expectations compared to more seasoned referees, or they may be eager to establish themselves within the academic community and build positive relationships with authors, leading to an inclination to accept manuscripts that meet basic requirements. Therefore, analyzing how experience with the peer-review process affects a referee’s propensity to accept manuscripts is ultimately an empirical question.

Our novel finding that a referee’s acceptance rate increases with the number of reports they complete contributes to this discussion. We find this effect to be incrementally significant after controlling for other available variables provided by Schwert (2021b), including the referee’s publication history in the *JFE*, the quality of their own published *JFE* papers, their university rank, and their prior experience serving on the *JFE* editorial board.

## Methodology and data

We collected our dataset from multiple sources. Information on referees’ acceptance/rejection recommendations for manuscripts they review for the *JFE* is reported in Table 13A in Schwert (2021b). This dataset covers a comprehensive set of 1,941 reviewers who provided at least one referee report for the *JFE* between 1994 and 2020 and aggregates the outcomes of 23,929 individual reviews prepared for the *JFE*. It contains information on the total number of referee reports completed for the *JFE* for every reviewer, as well as the total number of rejections and acceptances recommended in these reports. One report is recorded for each paper, regardless of the number of paper revisions. This treatment seems reasonable, given that prior research indicates that if one referee suggests acceptance, the likelihood of another referee agreeing increases only marginally, from 31 to 34% (Welch 2014). The dataset also provides information on the average turnaround time in days and an indicator variable that records whether a reviewer served on the *JFE* editorial board at any time between 1974 and 2020.

We matched these data with the information about the authors of the *JFE* papers provided in Table 5A in Schwert (2021b). Since Table 5A does not include first names, we mapped the two tables using the referees’ surnames and initials. We manually checked all cases where the mapping between the initials and first name(s) was ambiguous. Table 5A provides information for each author who published at least one article in the *JFE* between 1974 and 2020, including the total number of papers published in the *JFE*, the sum of coauthor shares in *JFE* papers, and the total number of citations of these *JFE* papers in the Web of Science Social Science Citation Index (SSCI). It also standardizes these citations according to citations per year since the paper was published, in terms of coauthor shares (where each of the *n* coauthors of a given paper receives 1/*n* credit), and according to a combination of citations per year since publishing and coauthor shares. Finally, the table contains information on the author’s affiliation when their most recent *JFE* paper was published.

We used the latter information from Table 5A to match the data on *JFE* referees and authors with the data on the academic institutions that employ them. Table 5A includes the ranking of institutions based on the number of publications in *JFE* and the number of citations of articles published in *JFE* by researchers affiliated with that institution. It also provides the aggregate number of published articles, coauthor shares, and citations by all university members in their conversions per author, per year, and per author-year.

We complemented this base dataset with additional data on referee characteristics. To obtain these data, we used a combination of several natural language chatbots, Google Scholar’s application programming interface (API), and manual collection. We collected information on the referee’s gender, origin (nationality and citizenship), the university where they completed their doctoral studies, and the field of their doctoral studies. We also collected information on referees’ total number of publications in Google Scholar, the total number of citations, their H-index (which specifies the number of *n* publications that are cited in Google Scholar at least *n* times), the total number of unique coauthors, and the number of articles published in the *JFE*. We provide detailed information on our data search using chatbots and the Google Scholar API in the Appendix. Furthermore, we hand-collected data on the overall annual rejection rates in the *JFE*.

We used a referee’s acceptance rate (*Accept*) as our dependent variable and defined it as a ratio of the number of recommendations to accept a manuscript to the total number of reports provided by a given referee during our sample period. We examined whether a referee’s acceptance rate is affected by his or her experience with the peer-review process in the *JFE*. We defined our main explanatory variable as the natural logarithm of the number of manuscripts a given referee had reviewed over his or her career (*#Reports*). In alternative specifications, we replaced this main variable of interest with an indicator, *More1Reps*, defined as ‘one’ for referees who graduated after 1994 and had prepared more than one referee report and ‘zero’ for those who graduated after 1994 and had prepared a single referee report. Correspondingly, we defined an indicator variable, *More3Reps,* where ‘one’ represented reviewers who graduated after 1994 and had provided more than three referee reports. We set *More1Reps* and *More3Reps* as ‘missing’ for referees who had completed their doctoral program before 1994. Furthermore, *More3Reps* was set to ‘missing’ for referees with two or three reports.

We used several additional explanatory variables to control for potential confounding effects. In our main tests, we included the *JFE* annual rejection rate averaged over the years during which the given researcher was active as a potential *JFE* referee (*RejectJFE*). We also used a dummy variable that indicates whether a given reviewer had served on the *JFE* editorial board at any time between 1974 and 2020 (*EditorJFE*). Furthermore, we used the natural logarithm of one plus the number of articles a given referee had published in the *JFE* between 1974 and 2020 (*#PubsJFE*) and the natural logarithm of the average annual number of times each of these publications was cited in the SSCI, scaled by the number of publications (*#CitsSSCI*). In addition, we included the negative of the natural logarithm of university rank based on the number of papers published in the *JFE* by authors affiliated with that university during the period from 1994 to 2020 (*UniRank*), where a higher number indicates a better-ranked institution. We also controlled for several measures extracted from Google Scholar: the natural logarithm of one plus the referee’s total number of publications in Google Scholar (*#PubsGS*), the natural logarithm of one plus the sum of referee’s citations in Google Scholar (*#CitsGS*), the natural logarithm of a referee’s H-index (*H-IndexGS*), the natural logarithm of one plus the number of a referee’s publications in the *JF* (*#PubsJF*), and the natural logarithm of the number of unique coauthors for all the publications listed on a given researcher’s Google Scholar profile website scaled by the number of years since PhD graduation (*#Coauths*). We winsorized all continuous variables at the top and bottom 1% of all observations.

We also used four measures of referees’ seniority in additional tests. We defined an indicator variable *Senior7y*, which equals ‘one’ for referees who completed their doctoral studies more than seven years before the start of our data sample period in 1994. The seven years correspond to the typical duration of the tenure track clock. Correspondingly, we defined *Junior7y* as equal to ‘one’ for referees who had completed their doctoral studies less than seven years before the end of our sample period. The former group of researchers was senior during all years of their presence in the data sample, while the latter group represented researchers who were still junior when our sample period ended. We defined similar measures, *Senior20y* and *Junior20y*, based on 20-year limits, as these represent approximately one-half of an active academic career after doctoral graduation.

Finally, given that prior research has investigated the impact of a referee’s gender (Card et al. 2020; Heidari et al. 2016), we also used an indicator variable, *Female,* to distinguish between male and female referees. We further set a dummy variable, *NonUS,* as equal to ‘one’ if their citizenship was not specified as “United States” or their nationality was not specified as “American” (or both). As a robustness check, we treated all referees whose citizenship included “United States,” “U.S.,” or “US”, or whose nationality was specified as “American” as the US referees (with either one of these conditions sufficient for the classification). In untabulated results, we observed that this modification of our definition did not materially affect our results, so we solely used this variable.

We used an indicator variable, *PrestigePhD,* to identify reviewers who graduated from PhD programs at prestigious universities. To identify universities that are commonly regarded as prestigious, we relied on two internationally recognized rankings of academic institutions: the Global Ranking of Academic Subjects (GRAS 2021)—also known as the Shanghai Ranking—which publishes world university rankings by academic subject, and the QS World University Rankings (QS 2024). Since the name *JFE* refers to two disciplines, we used information from two fields: (1) finance (GRAS 2021) and accounting and finance (QS 2024) and (2) economics (GRAS 2021) and economics & econometrics (QS 2024). Naturally, these four rankings substantially overlap; however, they are not quite identical. To increase the robustness of our classification of universities, we set an indicator variable, *PrestigePhD,* equal to ‘one’ for universities that appear in at least two of these four rankings and ‘zero’ otherwise. This procedure yielded the following list of prestigious universities (in alphabetical order): Columbia University, Harvard University, London School of Economics and Political Science, Massachusetts Institute of Technology, New York University, Princeton University, Stanford University, University of California Berkeley, University of Cambridge, University of Chicago, University of Oxford, University of Pennsylvania, and Yale University. When performing this classification, we also correctly classified the business schools of corresponding universities (e.g., “Stern” for New York University, “Booth” for the University of Chicago, and “Wharton” for the University of Pennsylvania).

We created categorical variables to represent the fields of specialization in reviewers’ doctoral studies. Specifically, we defined four categorical variables—*PhD_Finance*, *PhD_Economics*, *PhD_Accounting*, and *PhD_Law*—based on the following keywords: “Financ*,” “Economic*,” “Account*,” and “Law.” The *PhD_Management* categorical variable included keywords such as “Business,” “Manage*,” “Strateg*,” and “Marketing.” Additionally, we defined the *PhD_Maths* category using keywords such as “Mathematic*,” “Statistic*,” “Physic*,” and “Engineer*.” These categories are not mutually exclusive, except for “Economics,” which is explicitly separated from the others. For example, “Financial Economics” is classified under finance rather than economics, and “Business Economics” falls under management. Fields without any of these keywords were grouped into the *PhD_Other* indicator variable. Using finance as the baseline category, we included the remaining indicators in our regressions to evaluate their relative impact on acceptance rates. We collected data on all the mentioned variables for 1,718 referees, forming the final sample used in our regression analysis.

**Fig. 1:**
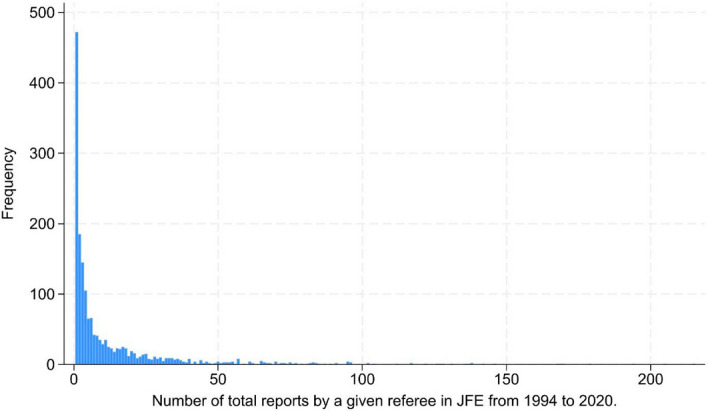
Histogram of the number of referee reports prepared by individual reviewers

Figure 1 illustrates the distribution of the number of referee reports prepared by individual reviewers. Not surprisingly, the distribution is highly skewed. Of the total of 1,718 referees, 472 (27%) prepared only a single referee report. In contrast, the most prolific referee prepared 215 reports, i.e., more than 50 times the median value. We addressed this issue by logarithmically transforming our key independent variable of interest (*#Reports*), and we clustered standard errors at the number of reports provided by a given referee. To further strengthen the confidence that our results are not driven by a few extreme observations, we also re-estimated our main regression using the subsample of data on referees within the interquartile range of report counts (i.e., referees with a minimum of two and a maximum of 19 reports). We discuss these results below.

Table 1 provides descriptive statistics for all the variables we used in our empirical analysis. For ease of interpretation, we report descriptive statistics for variables before their logarithmic transformation and winsorization. Table 1 shows that due to the skewness of the distribution of the number of referee reports prepared by individual reviewers, the mean value for the number of reports is 13, while the median value is only 4. Table 1 also shows that, on average, referees recommend accepting less than 7% of reviewed manuscripts. Interestingly, the acceptance rate is even lower than what would correspond to the average annual *JFE* rejection rate computed for the active years of the individual referees, which ranges from 85 to 91%. Considering only referees who completed their doctoral studies after 1994, 71% prepared more than one report, and 64% prepared more than three.

**Table 1:** Summary statistics

Variables	N	Mean	St. Dev	Minimum	Median	Maximum
*Accept*	1,718	0.066	0.093	0.000	0.000	0.500
#Reports*	1,718	13.014	22.932	1.000	4.000	215.000
*More1Reps*	1,287	0.713	0.453	0.000	1.000	1.000
*More3Reps*	1,024	0.639	0.481	0.000	1.000	1.000
*RejectJFE*	1,718	0.895	0.012	0.848	0.896	0.911
*AvgAge*	1,718	40.833	7.348	28.000	39.500	65.500
*Senior7y*	1,718	0.129	0.336	0.000	0.000	1.000
*Junior7y*	1,718	0.152	0.359	0.000	0.000	1.000
*Senior20y*	1,718	0.015	0.120	0.000	0.000	1.000
*Junior20y*	1,718	0.579	0.494	0.000	1.000	1.000
*Female*	1,718	0.159	0.366	0.000	0.000	1.000
*NonUS*	1,718	0.562	0.496	0.000	1.000	1.000
*PrestigePhD*	1,718	0.499	0.500	0.000	0.000	1.000
*PhD_Finance*	1,718	0.531	0.499	0.000	1.000	1.000
*PhD_Economics*	1,718	0.348	0.476	0.000	0.000	1.000
*PhD_Accounting*	1,718	0.050	0.218	0.000	0.000	1.000
*PhD_Management*	1,718	0.073	0.261	0.000	0.000	1.000
*PhD_Law*	1,718	0.004	0.064	0.000	0.000	1.000
*PhD_Maths*	1,718	0.012	0.107	0.000	0.000	1.000
*PhD_Other*	1,718	0.008	0.090	0.000	0.000	1.000
*EditorJFE*	1,718	0.040	0.196	0.000	0.000	1.000
#PubsJFE*	1,718	0.081	0.100	0.000	0.056	1.000
UniRank*	1,105	59.836	76.724	1.000	32.000	601.000
#Coauths*	1,718	1.688	1.584	0.000	1.220	14.900
#PubsGS*	1,718	2.661	2.496	0.200	1.976	35.333
#PubsJF*	1,718	0.123	0.711	0.000	0.074	29.023
*H-IndexGS*	1,718	1.083	0.644	0.042	0.933	9.000
#CitsGS*	1,718	392.519	687.454	0.208	195.897	11,098.590
#CitsSSCI*	1,718	78.429	128.496	0.000	42.550	2,282.167

*denotes variables that are log-transformed for further regression analyses

The descriptive statistics further indicate that the average age of a referee in our sample is 41 years, or 11 years since PhD graduation. Approximately 13% of the referees had more than seven years of experience for all their active years in our sample, and approximately 15% completed their doctoral studies less than seven years before the end of our sample period. Approximately 16% of the reviewers are female, approximately 56% have a non-US origin, and approximately half graduated from doctoral programs at one of the 13 most prestigious universities identified by the procedure outlined above. More than half of the referees in our sample earned their doctorates in finance, while over a third graduated from doctoral programs in economics. Additionally, approximately 4% served on the *JFE* editorial board at some point between 1974 and 2020.

In terms of scholarly output, referees in our sample list an average of approximately 2.7 Google Scholar papers per year of their career, which is close to the median value of 2.0. Furthermore, referees published an average of 0.12 papers per year of their career in the *JF* and 0.08 papers per year of their career in the *JFE*. The referees have an H-index scaled by the number of years of their career of approximately 1. The distribution of the number of Google Scholar citations per year of a referee’s career is highly skewed; the mean value of 393 is much higher than the median of 196. Similarly, the average number of normalized annual citations in the SSCI has a mean of 78 and a median of 43. The mean number of coauthors of Google Scholar papers scaled by the number of years of the reviewers’ academic career is 1.7.

## Results and discussion

**Fig. 2:**
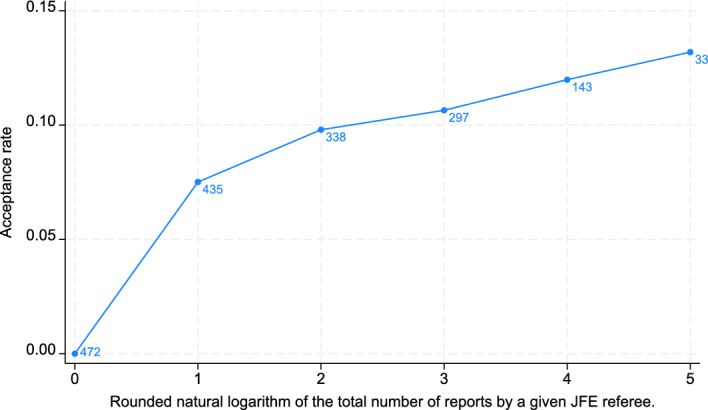
Average acceptance rate computed as the number of recommendations to accept a reviewed manuscript divided by the total number of recommendations by a given referee. The blue numbers at the individual values indicate the number of referees in a given category

We start our empirical analysis of the association between referees’ experience with the peer-review process and their likelihood of recommending manuscript acceptance by visualizing the association between the two constructs. Figure 2 displays the acceptance rate for different categories of referee report numbers that we log-transformed and rounded to the nearest integer number. The figure shows that referees’ propensity to recommend acceptance monotonously increases across the individual categories, representing the level of their experience with the peer-review process in the *JFE*. Thus, Fig. 2 provides preliminary evidence that the propensity to accept reviewed manuscripts increases with peer-review process experience. Interestingly, none of the 472 referees who prepared just one referee report suggested accepting the manuscript they reviewed.

We draw similar conclusions based on our regression analysis, which is reported in Table 2. We used a referee’s acceptance rate (*Accept*) as our dependent variable, defined as a ratio of the number of recommendations for manuscript acceptance to the total number of reports provided by a given referee during our sample period. We used #*Reports* as our main independent variable of interest, which proxies for the referee’s experience with the peer-review process in the *JFE*. Due to its skewed distribution, it was log-transformed. We also clustered standard errors at the number of reports provided by a given referee. In nontabulated results, we observe that when standard errors are not clustered, the results are qualitatively similar and numerically stronger. We used several other control variables that may be associated with referees’ propensity to accept manuscripts and examined them separately and in combination with other variables. This approach facilitated the evaluation of the robustness of our main findings to the inclusion of various sets of control variables.

**Table 2:** Baseline results

	(1)	(2)	(3)	(4)	(5)	(6)	(7)	(8)
Variables	Accept	Accept	Accept	Accept	Accept	Accept	Accept	Accept
*#Reports*	0.032***							
	(7.92)							
*RejectJFE*		− 1.504***						
		(− 6.21)						
*AvgAge*			0.111***					
			(6.18)					
*EditorJFE*				0.073***				
				(3.74)				
*#PubsJFE*					0.138			
					(1.29)			
*UniRank*						0.016***		
						(5.33)		
*#Coauths*							0.016***	
							(3.02)	
*#PubsGS*								0.027***
								(3.51)
*#PubsJF*								
*H-IndexGS*								
*#CitsGS*								
*#CitsSSCI*								
Intercept	0.015	1.412***	− 0.346***	0.063***	0.056**	0.120***	0.052***	0.034**
	(1.11)	(6.12)	(− 6.34)	(3.13)	(2.15)	(8.99)	(2.67)	(2.53)
Cluster	YES	YES	YES	YES	YES	YES	YES	YES
Adjusted R^2^	0.230	0.038	0.041	0.024	0.014	0.038	0.004	0.015
N	1,718	1,718	1,718	1,718	1,718	1,718	1,718	1,718

	(9)	(10)	(11)	(12)	(13)	(14)	(15)
Variables	Accept	Accept	Accept	Accept	Accept	Accept	Accept
*#Reports*					0.036***	0.034***	0.038***
					(9.51)	(7.95)	(11.05)
*RejectJFE*					0.528	0.170	0.285
					(0.84)	(0.30)	(0.49)
*AvgAge*					0.094**	0.087**	0.091**
					(1.99)	(1.96)	(2.00)
*EditorJFE*					-0.008		–0.004
					(-0.84)		(–0.38)
*#PubsJFE*					–0.196***		–0.199***
					(–2.92)		(–2.92)
*UniRank*					0.005*		0.004**
					(1.94)		(2.20)
*#Coauths*					0.015**		–0.001
					(2.05)		(–0.19)
*#PubsGS*						0.012	0.014*
						(1.54)	(1.72)
*#PubsJF*	0.107					–0.100*	–0.076
	(1.32)					(–1.83)	(–1.59)
*H-IndexGS*		0.066***				0.049***	0.048**
		(6.67)				(2.66)	(2.28)
*#CitsGS*			0.020***			–0.008**	–0.006
			(11.16)			(–2.23)	(–1.56)
*#CitsSSCI*				0.007***		–0.003	–0.005**
				(3.66)		(–1.56)	(–1.97)
Intercept	0.056**	0.020	–0.041**	0.039*	–0.793	–0.445	–0.548
	(2.17)	(1.31)	(–2.17)	(1.78)	(–1.11)	(–0.69)	(–0.83)
Cluster	YES	YES	YES	YES	YES	YES	YES
Adjusted R^2^	0.010	0.031	0.060	0.008	0.265	0.258	0.281
N	1718	1718	1718	1718	1718	1718	1718

Standard errors are clustered at the number of referee reports. ***, **, and * denote significance levels at the 1%, 5%, and 10% levels, respectively

Table 2 demonstrates consistent evidence of the importance of #*Reports* in explaining the variation in *Accept*. When #*Reports* is used as a sole explanatory variable (column 1), its slope coefficient is positive (0.032) and statistically significant (*t*-stat: 7.92). We also examined the importance of several other potentially relevant explanatory variables. Consistent with our expectations, we observe that the average annual rejection rate at the *JFE* in years of a given referee’s active career (*RejectJFE*) is significantly negatively associated with *Accept* (*t*-stat: − 6.21). The variable controls for the time-series variation in the overall strictness of the journal editors, which may affect referees’ recommendations. In nontabulated results, we observe that the annual rejection rates increased over time, from 85% in 1994 to 91% in 2020. This result further suggests that referees who prepare their reports in years when the journal rejects a larger proportion of manuscripts are less likely to recommend acceptance. In a multivariate setting (columns 13–15), the effect of this variable is subsumed by other explanatory variables. Importantly, including this additional control variable does not seem to diminish the explanatory power of the main variable of interest (*#Reports*), which remains significant at the 1% level.

To further investigate the effect of time-series variation on *RejectJFE*, we re-estimated our results using data exclusively from reviewers who graduated after 2012 as a sensitivity analysis. By that year, the JFE rejection rate had risen to 90% and stabilized. The untabulated results confirm that *RejectJFE* is not statistically significant in the multivariate analysis. This indicates that for the period 2012–2020, any residual variation in the journal’s annual rejection rate does not significantly explain the variation in referees’ propensity to recommend acceptance. Meanwhile, our primary finding regarding the importance of *#Reports* remains statistically significant for this subsample (n = 295), strengthening our confidence that although publication standards have tightened over time, this trend does not overshadow our main result.

We also consider the possibility that the propensity to recommend manuscript acceptance may be associated with referees’ general seniority. To control for seniority and disentangle it from the specific effect of their direct experience with the peer-review process, we include an additional control variable—a referee’s average age over their active years during the sample period, defined in relation to their PhD graduation year (*AvgAge*). As expected, the slope coefficient on this variable is positive and statistically significant in the univariate (column 3) and multivariate (columns 13–15) settings. This suggests that older referees are, ceteris paribus, more likely to recommend accepting the manuscripts they review. Nevertheless, including *AvgAge* as an additional control variable does not materially affect the explanatory power of *#Reports*.

We assume that the selection of a referee is primarily based on how well their area of expertise aligns with the manuscript’s topic. However, we acknowledge the possibility that the selection may also be influenced by *JFE* Editors’ familiarity with potential referees. We considered four empirical variables that proxy for likely familiarity. First, we argue that journal editors are more likely to be acquainted with potential referees when these referees have served on the journal’s editorial board. We defined an indicator variable, *EditorJFE*, equal to ‘one’ for referees who served on the *JFE* editorial board between 1974 and 2020 and ‘zero’ otherwise. Second, we expect the *JFE* Editors to be more familiar with researchers who successfully publish articles in the *JFE,* so we considered the number of articles that a given referee had published in the *JFE* (*#PubsJFE*). Third, due to their interactions at research seminars and similar events, we expect editors at prominent finance journals to be better acquainted with potential referees working at leading universities, and therefore, we used the standing of the universities employing the referees as an additional control variable (*UniRank*). Finally, we expect journal editors to be better acquainted with potential reviewers who have an extensive professional network and thus control for the number of unique coauthors (*#Coauths*).

Table 2 shows that in a univariate setting (columns 4–8), all four of the above proxies of familiarity between journal editors and potential referees are positively associated with *Accept*, but the association is only statistically significant for three of them (*EditorJFE*, *UniRank*, and *#Coauths*). Nevertheless, this association is not robust to the inclusion of additional controls. In a multivariate setting (column 15), only *UniRank* remained significantly positively associated with *Accept*. Hence, we observe limited consistent evidence on the relevance of familiarity proxies for explaining the variation in *Accept*.

Table 2 also displays the effect of several proxies that are likely to capture the depth of academics’ research expertise. We argue that due to the “publish or perish” imperative, research expertise is likely correlated with the number and quality of publications and the intensity with which these publications are cited. We considered five variables that capture these characteristics: (i) the number of a researcher’s Google Scholar publications (*#PubsGS*); (ii) the number of articles published in the *JF* (*#PubsJF*), arguably the most prestigious finance journal; (iii) the referee’s Google Scholar H-index (*H-indexGS*); (iv) the number of citations in Google Scholar (*#CitsGS*); and (v) the normalized citations in the SSCI (*#CitsSSCI*). The results in columns (8–12) show that in a bivariate setting, all but one of these variables (*#PubsJF*) have at least a marginally significant positive association with the acceptance rate. However, for three of the five variables, the slope coefficient switches sign in the multivariate setting. Furthermore, the H-index is the only variable that retains its significance in the expected direction when used in combination with all the other control variables (columns 14–15).

Combined, the empirical evidence on the relevance of the proxies for familiarity between journal editors and potential referees and of proxies for referees’ research expertise is not consistent for the individual measures or across various regression specifications. Nevertheless, including these measures as additional control variables in regression specifications does not compromise the ability of our main variable of interest, *#Reports,* to explain the variation in the referee’s propensity to recommend acceptance. The untabulated results verify that this conclusion about the statistically significant positive association between *#Reports* and *Accept* also extends to any pair of explanatory variables, including *#Reports* together with one of the ten above-discussed control variables. In fact, the empirical evidence on *#Reports* strengthens in the multivariate setting as the slope coefficient increases from 0.032 (univariate) to 0.038 (multivariate), and its statistical significance (*t*-stat) strengthens from 7.92 (univariate) to 11.05 (multivariate). We thus conclude that our main finding that the propensity of referees to recommend acceptance increases with their experience of the peer-review process is remarkably robust to the inclusion of these additional control variables.

We conducted several sensitivity and robustness tests. As illustrated in Fig. 1 and Table 1, the distribution of the number of reports prepared by individual referees is highly skewed. To mitigate this problem, we log-transform the *#Reports* measure. We also clustered standard errors at the number of reports provided by a given referee. To further strengthen confidence that our results are not driven by a few extreme observations, we first narrowed our analysis to referees within the interquartile range of report counts (i.e., referees with a minimum of two and a maximum of 19 reports). We re-estimate all our main results based on this subsample and report the results in Table 3. Naturally, using data only for the interquartile range not only lowers the number of available observations from 1,718 to 919 but also reduces the variability of our independent variable, impairing the power of our tests. Table 3 shows that our main result remains significant, even with this reduced statistical power. In the multivariate setting, the slope coefficient on *#Reports* remains positive and statistically significant at more than the 1% level. This increases the confidence that our main results are not driven by peculiar characteristics at either end of the distribution of reviewers. As a sensitivity check, we further included only referees with at least 10 and no more than 100 reports. In both cases, we find that the regression coefficients in Table 2 retain their signs and significance levels (results not tabulated).

**Table 3:** Sensitivity tests: Interquartile results

	(1)	(2)	(3)
Variables	Accept	Accept	Accept
*#Reports*	0.045***	0.043***	0.050***
	(2.84)	(2.76)	(3.33)
*RejectJFE*	0.865	0.400	0.725
	(0.76)	(0.38)	(0.73)
*AvgAge*	0.165**	0.170**	0.169***
	(2.35)	(2.42)	(2.66)
*EditorJFE*	0.069***		0.080***
	(2.76)		(2.68)
*#PubsJFE*	− 0.299***		− 0.304***
	(− 3.70)		(− 3.70)
*UniRank*	0.005*		0.005*
	(1.69)		(1.92)
*#Coauths*	0.019*		− 0.004
	(1.86)		(− 0.38)
*#PubsGS*		0.019*	0.019*
		(1.79)	(1.95)
*#PubsJF*		− 0.179**	− 0.168**
		(− 2.30)	(− 2.38)
*H-IndexGS*		0.068**	0.066**
		(2.38)	(2.11)
*#CitsGS*		− 0.012**	− 0.007
		(− 2.01)	(− 1.23)
*#CitsSSCI*		− 0.002	− 0.006*
		(− 0.94)	(− 1.71)
Intercept	− 1.354	− 0.956	− 1.227
	(− 1.05)	(− 0.79)	(− 1.08)
Cluster	YES	YES	YES
Adjusted R^2^	0.145	0.139	0.175
N	919	919	919

Standard errors are clustered at the number of referee reports. ***, **, and * denote significance levels at the 1%, 5%, and 10% levels, respectively

Second, we recognize that the propensity of referees to recommend accepting reviewed manuscripts may be affected by their seniority. To address this issue, we controlled for the average age of individual referees in our sample, defined relative to the year of their graduation from their doctoral program. To further examine the impact of referees’ general seniority and disentangle it from the specific effect of their direct experience with the peer-review process, we defined two companion indicator variables for researchers who were either senior or junior throughout their presence in our sample period. Specifically, we set *Senior7y* equal to ‘one’ for referees who completed their doctoral studies more than seven years before the start of our data sample period in 1994. The seven years correspond to the typical duration of the tenure track clock. Our results remain qualitatively similar when considering six or eight years as the standard tenure track duration (not tabulated). Correspondingly, we defined *Junior7y* as equal to ‘one’ for referees who had completed their doctoral studies less than seven years before the end of our sample period. The former group of researchers was senior for all the years of their presence in the sample, while the latter group represented researchers who were still junior at the end of the sample period. We defined similar measures, *Senior20y* and *Junior20y,* based on 20-year limits, representing approximately one-half of an active academic career after doctoral graduation. We argue that if general seniority rather than direct experience with the peer-review process drives our results, including these measures would subsume our main result on the number of referee reports prepared by a given referee.

These sensitivity results are reported in Table 4 and indicate that in a bivariate setting (columns 1–2), more senior referees are likelier, and more junior referees are less likely to recommend acceptance. The results in the first two columns have the expected sign, and three of the four coefficients are statistically significant. Nevertheless, this result disappears in the multivariate setting once we include *#Reports* and additional control variables in the regression specifications in columns (3–4). In these full regression specifications, three of the four coefficients on the juniority/seniority indicators are statistically insignificant, and the remaining one is statistically significant in the opposite direction relative to the predictions. In contrast, our main result on the positive impact of *#Reports* remains statistically significant in the expected direction. These results suggest that even though general seniority, when used as the only explanatory variable, plays some role in the propensity of referees to recommend acceptance, its impact is dominated by the effect of the direct experience with the peer-review process that is approximated by *#Reports*. We thus conclude that these results further support our main proposition of a positive association between experience with the peer-review process and the propensity to recommend accepting reviewed manuscripts.

**Table 4:** Sensitivity tests: Seniority results

	(1)	(2)	(3)	(4)
Variables	Accept	Accept	Accept	Accept
*#Reports*			0.038***	0.038***
			(10.49)	(10.80)
*Senior7y*	0.031***		0.009	
	(3.78)		(1.16)	
*Junior7y*	− 0.027***		− 0.004	
	(− 7.20)		(− 0.83)	
*Senior20y*		0.027		0.010
		(1.01)		(0.52)
*Junior20y*		− 0.025***		0.025***
		(− 6.39)		(2.61)
*RejectJFE*			− 0.755**	− 1.809***
			(− 2.06)	(− 2.80)
*EditorJFE*			− 0.004	− 0.005
			(− 0.32)	(− 0.44)
*#PubsJFE*			− 0.198***	− 0.201***
			(− 2.88)	(− 2.94)
*UniRank*			0.004**	0.005**
			(2.18)	(2.35)
*#PubsJF*			− 0.075	− 0.076
			(− 1.63)	(− 1.60)
*H-indexGS*			0.062**	0.063***
			(2.56)	(2.70)
*#CitsGS*			− 0.006	− 0.006*
			(− 1.45)	(− 1.68)
*#CitsSSCI*			− 0.005**	− 0.005**
			(− 2.00)	(− 1.99)
Intercept	0.066***	0.080***	0.722**	1.653***
	(3.47)	(4.02)	(2.11)	(2.82)
Cluster	YES	YES	YES	YES
Adjusted R^2^	0.026	0.020	0.278	0.282
N	1,718	1,718	1,718	1,718

Standard errors are clustered at the number of referee reports. ***, **, and * denote significance levels at the 1%, 5%, and 10% levels, respectively

Third, we recognize that due to the limitations of our dataset, which is aggregated at the level of individual referees, we cannot specifically measure referees’ prior experience with the peer-review process before they prepare a given referee report. Nevertheless, we directly identified a lack of prior experience in cases where our dataset permitted this (i.e., for reviewers who completed their doctoral studies after the beginning of our sample period [i.e., 1994] and at the same time prepared only a single referee report throughout their career). We can be confident that these reviewers prepared their report without any prior experience with the *JFE* peer-review process. We used this subset of referees as a baseline group. We argue that, compared to this group, our data on the remaining reviewers—those who graduated after 1994 and completed more than one referee report—represent a combination of recommendations from experienced and inexperienced reviewers. However, we acknowledge that this subsample does not exclusively represent experienced reviewers, as it includes their first referee reports, completed before they had gained any prior experience. Since we cannot distinguish between the first and subsequent referee reports for a given reviewer, data for this latter group are based on a mix of experienced and inexperienced reviews. Nevertheless, we also note that the proportion of experienced reviews increases with the total number of referee reports a given reviewer prepared throughout his or her career.

To directly compare reports by referees confirmed to have had no prior experience with the *JFE* peer-review process to those who may have had some experience, we substituted our primary explanatory variable, *#Reports*, with a binary variable, *More1Reps*. This indicator was set to ‘one’ for referees who graduated after 1994 and completed more than one referee report. We set *More1Reps* to ‘zero’ for referees who graduated after 1994 and prepared a single report throughout their career. We set the measure to ‘missing’ for the remaining reviewers. To increase the likelihood that the reports in the latter group were performed by referees with prior experience with the *JFE* peer-review process, we defined another indicator variable, *More3Reps*, which we set to ‘missing’ for reviewers who had prepared two or three reports. *More3Reps* is thus equal to ‘one’ only for reviewers for whom the inexperienced first report constitutes no more than 25% of their track record.

Table 5 presents the empirical results of these regressions. Despite the weakened statistical power of these tests, the slope coefficients remain positive and statistically significant at a better than 1% level for *More1Reps* and *More3Reps* in columns 2 and 3, respectively. We conclude that by performing an empirical test distinguishing between clearly inexperienced and potentially experienced reviewers in a way our dataset permits, we observe similar results to our main findings. These results further support our proposition that experienced referees are more likely to recommend the acceptance of reviewed manuscripts.

**Table 5:** Sensitivity tests: Referee experience

	(1)	(2)	(3)
Variables	Accept	Accept	Accept
*#Reports*	0.039***		
	(12.33)		
*More1Reps*		0.074***	
		(3.69)	
*More3Reps*			0.104***
			(16.45)
Intercept	− 2.168	− 2.621	− 1.862
	(− 1.55)	(− 1.48)	(− 1.20)
Controls	YES	YES	YES
Cluster	YES	YES	YES
Adjusted R^2^	0.283	0.217	0.379
N	1287	1287	1024

Controls include the same set as in Table 2. Standard errors are clustered at the number of referee reports. ***, **, and * denote significance levels at the 1%, 5%, and 10% levels, respectively

As a final robustness test, we complemented our main analysis by collecting information on several referee-related characteristics that do not change over time throughout their academic career (i.e., referees’ gender, origin, alma mater of their doctoral studies, and the field of their doctoral studies) and investigated whether the reviewer-specific component of the acceptance rate can be attributed to these characteristics.

Table 6 shows our regression results incorporating these characteristics. When these characteristics are used as the only explanatory variable in each model (columns 1–4), we observe that they are significantly associated with *Accept*. For example, female referees and non-US referees are less likely to recommend acceptance. In contrast, referees who graduated from PhD programs at prestigious universities are more likely to recommend acceptance. Considering the various disciplines of referees’ doctoral training, we observe marginally significant support in some specifications for referees in the fields of economics, management, and mathematics, who are more likely to recommend the acceptance of manuscripts relative to reviewers with a PhD in finance (our baseline category). Conversely, we find a weak indication that referees in law and accounting tend to be less likely to recommend acceptance. Nevertheless, among these personal-level characteristics, only *PhD_Maths* is statistically significant at the conventional 5% level when combined with the control variables used in our main results, as reported in Table 2. We thus conclude that only limited evidence on the relevance of these characteristics has been provided.

**Table 6:** Sensitivity tests: Referee characteristics

	(1)	(2)	(3)	(4)	(5)	(6)	(7)	(8)	(9)	(10)
Variables	Accept	Accept	Accept	Accept	Accept	Accept	Accept	Accept	Accept	Accept
*#Reports*						0.038***	0.038***	0.038***	0.038***	0.037***
						(11.03)	(11.06)	(10.77)	(10.92)	(10.79)
*Female*	− 0.013***				− 0.013***	− 0.003				− 0.003
	(− 3.18)				(− 3.26)	(− 0.73)				(− 0.87)
*NonUS*		− 0.015***			− 0.013**		0.001			0.002
		(− 3.03)			(− 2.30)		(0.37)			(0.50)
*PrestigePhD*			0.026***		0.022***			0.008**		0.006*
			(5.91)		(4.75)			(2.18)		(1.79)
*PhD_Economics*				0.022**	0.016				0.012**	0.011*
				(2.01)	(1.36)				(2.08)	(1.86)
*PhD_Accounting*				− 0.011	− 0.012				− 0.007	− 0.005
				(− 1.23)	(− 1.20)				(− 0.65)	(− 0.58)
*PhD_Management*				0.024*	0.017				0.018	0.017
				(1.72)	(1.20)				(1.60)	(1.59)
*PhD_Law*				− 0.027	− 0.044*				− 0.022	− 0.024
				(− 1.26)	(− 1.66)				(− 0.93)	(− 1.01)
*PhD_Maths*				0.049*	0.053*				0.046**	0.046**
				(1.94)	(1.95)				(2.22)	(2.14)
*PhD_Other*				− 0.027	− 0.035**				− 0.019	− 0.020
				(− 1.54)	(− 2.02)				(− 1.01)	(− 1.04)
Intercept	0.068***	0.074***	0.053***	0.057***	0.058***	− 0.557	− 0.565	− 0.562	− 0.600	− 0.654
	(3.37)	(3.85)	(2.74)	(3.48)	(3.78)	(− 0.84)	(− 0.88)	(− 0.85)	(− 0.91)	(− 1.02)
Controls	NO	NO	NO	NO	NO	YES	YES	YES	YES	YES
Cluster	YES	YES	YES	YES	YES	YES	YES	YES	YES	YES
Adjusted R^2^	0.002	0.006	0.020	0.017	0.036	0.281	0.281	0.283	0.287	0.287
N	1718	1718	1718	1718	1718	1718	1718	1718	1718	1718

Controls include the same set as in Table 2. Standard errors are clustered at the number of referee reports. ***, **, and * denote significance levels at the 1%, 5%, and 10% levels, respectively

The last five columns in Table 6 further show that including these additional personal-level characteristics in the regression specifications materially affects neither the magnitude nor the statistical significance of the slope coefficient of our main variable of interest, *#Reports*. Thus, we conclude that while we observe only a limited effect of the referee’s personal-level characteristics in a multivariate setting, our main results are robust to the inclusion of these characteristics as control variables. These findings contribute to the discussion of gender in research, following the Sex and Gender Equity in Research (SAGER) guidelines designed to promote systematic reporting of the potential impact of gender differences in empirical research (Heidari et al. 2016).

## Conclusion

We analyze whether and to what extent referees’ experience with refereeing for and publishing in the *JFE* affects their propensity to recommend accepting a manuscript under review. Our results demonstrate that referees who review more manuscripts are consistently and robustly more inclined to recommend accepting manuscripts for publication. We acknowledge that a referee’s decision to accept or reject a manuscript further depends on factors such as its quality, contributions to the research literature, and the credibility of the empirical evidence presented (Baghestanian and Popov 2018). Nevertheless, we identify an additional factor that is related to the recommendation. Our results suggest that more seasoned and experienced referees may be able to better evaluate the strengths and weaknesses of papers under review and guide the authors through constructive suggestions toward eventual paper acceptance, whereas less experienced referees may feel more pressure, have less confidence, and be more cautious in their judgment, leading them to reject submissions more frequently than their more experienced colleagues.

By documenting the association between referees’ recommendations in the peer-review process and their experience of the process, our study yields several valuable theoretical and practical implications. The peer-review process is an important, commonly used procedure for evaluating the quality of scientific manuscripts and research proposals (Jefferson et al. 2002; Shopovski et al. 2020). The outcome of peer review is vital in shaping the development of established knowledge in all scientific disciplines. It is also used as a basis for deciding how society allocates scarce resources to conduct future research. Given its importance, it is socially beneficial to improve our understanding of the factors affecting the outcome of the peer-review process.

Agathokleous (2021) refers to reviewers as “sentinels of science” and argues that editors’ decisions largely depend upon reviewers’ recommendations. He also points out that reviewers’ recommendations sometimes do not align with their comments to authors and advise against such a “two-faced policy.” Consistency and precision in the peer-review process promote efficient research conduct. We were unable to observe reviewer comments in the data collected for this study. Thus, potential discrepancies between reviewers’ recommendations and their comments to authors represent a possible limitation.

In terms of theory, our study supports the socioemotional selectivity theory proposed by Carstensen et al. (1999), which suggests that as people grow older, they become more selective in their social relationships, prioritizing meaningful interactions. This theory may explain why more experienced referees tend to be more supportive of other authors. Our results are also consistent with the interdependence theory summarized by Van Lange and Balliet (2014), which posits that individuals weigh the costs and benefits of cooperation versus competition in their interactions with others. Our study provides empirical evidence on the applicability of these theories to a peer-review process setting, where they have not yet been tested. Given the centrality of the peer-review process in guiding the efficient allocation of resources and conduct of research, it constitutes an important setting in which to assess the relevance of these theories and the breadth of their applicability.

In terms of practical implications, our study identifies that referees’ experience with the peer-review process is a vital determinant of their recommendations. We provide extensive and consistent evidence establishing that this association prevails even after considering a host of other potentially relevant explanatory variables. These findings are informative to all those involved in any kind of peer-review process. Reviewers of manuscripts may consider this finding when benchmarking and calibrating their evaluations. Journal editors may wish to consider our findings when establishing their editorial policies and in choosing reviewers for manuscripts. We report that the *JFE* Editors who requested reviews from inexperienced referees received a recommendation to reject the reviewed manuscript in 100% of cases. This finding suggests a strong association between referee experience and the likelihood of recommending rejection. Professional bodies that set the parameters of peer-review processes and provide oversight of scientific journals and foundations may wish to consider our findings to appreciate the importance of diversity in terms of the prior experience of referees who evaluate scientific manuscripts and research proposals. Our results may also underscore the importance of benchmarking between more and less experienced referees by those who make the ultimate decision and motivate them to consider providing training and mentorship programs for less experienced referees.

Our study is constrained by the limited granularity of the main dataset, which was sourced from Schwert (2021b) and is aggregated at the level of individual reviewers. More granular data would enable a finer analysis to provide stronger conclusions about the mechanisms underlying the pattern we document here. We call for future research based on more detailed data to explore these findings further.

## Data Availability

The datasets used and analyzed during the current study are available from the corresponding author on reasonable request.

## Appendix

For our empirical analysis, we used data on individual referees’ characteristics. Specifically, we gathered information on each referee’s gender, origin (nationality and citizenship), year of graduation from their doctoral program, the university where they earned their doctorate, and the field of study for their doctoral research. Additionally, we collected data on the total number of publications listed in Google Scholar, total citations, H-index (indicating the number *n* of publications with at least *n* citations), total number of unique coauthors, and the number of articles published in the *Journal of Finance (JF)*. As this information is dispersed across multiple sources, manually compiling it would be labor intensive and potentially cost prohibitive. To streamline the data collection process, we leveraged a combination of natural language chatbots and Google Scholar’s application programming interface (API). The appendix provides a detailed summary of the methodology.

We collected the data in two rounds. This approach was driven partly by the distinct sources from which each dataset was obtained and partly to mitigate “mid-prompt chatbot fatigue,” which can occur with lengthy prompts. In the first round, we gathered personal information that is typically available from referees’ personal websites, curriculum vitae (CVs), and LinkedIn profiles. This includes details such as gender, origin (nationality and citizenship), graduation year from their doctoral program, the university where they earned their degree, and the field of their doctoral studies. In the second round, we collected data from the referees’ Google Scholar profile pages.

For the first search, we used several natural language chatbots (details provided below), accessed via their dedicated APIs or Poe’s API (https://poe.com/). We developed Python code to iterate through the list of referees’ full names. Based on our experience, it is beneficial to provide a detailed system prompt describing the task and a concise user prompt specifying the professor’s name. Optimal results were achieved by including an example of the desired response format in the system prompt. When using dedicated APIs, we set the temperature to 0.1 to minimize creativity in the responses and limit the maximum number of tokens to 100 to ensure concise outputs. For the first round of data collection, we used the following combination of prompts.

System prompt: “*Your task is to provide detailed and accurate information about a finance professor. Please ensure your response is structured clearly and includes the following elements: Full Name: Provide the complete name of the Professor. Gender: Specify whether the Professor is male or female. Nationality: Indicate the nationality of the Professor. Country of Citizenship: Name the country where the Professor holds citizenship. Year of Doctoral Completion: State the year when the Professor completed their PhD program (which may be abbreviated as PhD or PhD). Field of Research: Identify the field in which the Professor earned their doctorate (e.g., Finance, Economics, Mathematics). University Attended for Doctorate: Mention the university where the Professor completed their PhD Your response should be concise and formatted as a single line of text, with each value separated by semicolons. For example: Andrew Abel; Male; American; United States; 1978; Economics; Massachusetts Institute of Technology. Ensure that all information is factual and drawn from reliable sources. Take more time to process the prompt if necessary.*”

User prompt: “*Retrieve information about finance professor ‘FULL_NAME’. Respond with a single line of text, values separated by semicolons: Full Name; Gender; Nationality; Country of Citizenship; Year of Doctoral Completion; Field of Research; University Attended for Doctorate. No extra text.*”

For the second round of data collection, we used the following combination of prompts.

System prompt: “*Your task is to provide detailed and accurate information about a finance professor. Search for the professor’s personal profile page on Google Scholar (*https://scholar.google.com/*). If the Google Scholar profile page exists, extract the following information: 1) Full name: The professor’s full name as stated on the Google Scholar profile. 2) Number of citations: The total number of citations as stated on the Google Scholar profile. 3) H-index: The professor’s H-index as stated in the publication statistics on Google Scholar profile. 4) Number of unique coauthors: Count the number of unique coauthor names on all of the professor’s publications listed on Google Scholar. 5) The total number of publications published in any journal. 6) Number of publications published in the ‘Journal of Finance’, the attribute ‘journal’ of a given publication must exactly match ‘Journal of Finance’ or ‘the Journal of Finance’. The capitalization of words in the journal names does not matter. Your answer should be short and reported in a single line of text, with individual values separated by semicolons. For example: Greg Duffee; 8200; 24; 6; 52; 6. Do not include commas within individual values. Do not include any commentary, citations (e.g., references in brackets), or explanations. Do not include any citations, references, or bracketed text (e.g., [1], [2]). Make sure all information is factual and comes from the Google Scholar profile. Take more time to execute the prompt if necessary.*”

User prompt: “*Retrieve information about finance professor ‘FULL_NAME’. Respond with a single line of text, values separated by semicolons: Full Name; Number of Citations in Google Scholar; H-index in Google Scholar; Number of Unique Co-Authors in Publications in Google Scholar; Total Number of Publications in Google Scholar; Number of Publications in Journal of Finance. No extra text.*”

We determined that maximizing the precision of the collected data requires the chatbot to perform a web search before addressing the prompt. However, not all chatbots conduct a web search before generating a response. For instance, ChatGPT in versions 4.0 or 4.0 Mini automatically performs a web search when accessed through the graphical user interface (GUI), but this functionality is not available when accessed via the API. As a result, the accuracy of responses provided by ChatGPT differs significantly depending on whether it is accessed through the GUI or the API.

We obtained the best results with the Perplexity API using the “llama-3.1-sonar-large-128 k-online” chatbot model. We also obtained fairly reliable results using “gemini-1.5-flash-search” accessed through the Poe API and somewhat reliable results using “web-search” and “solar-pro,” both accessed through the Poe API. In contrast, we failed to obtain useful responses using various versions of the following chatbots: claude-3.5-sonnet, command-r-plus, grok, solar-pro, and neeva. In addition, we obtained unreliable results when using AutoIt to automate sending requests to ChatGPT 4o. We failed to collect data for more than a few referees using Playwright, Selenium, or Axiom.ai, which theoretically may be used to automate sending requests to ChatGPT 4o. We presume (but cannot state with certainty) that this is mainly because ChatGPT (OpenAI) restricts data collection in larger batches.

For the first round of data collection, we collected the data on all referees in our sample using (1) Perplexity API, (2) Gemini by Poe API, and (3) Web-search by Poe API. We applied the following rules. First, if the information about a given referee generated by Perplexity was complete and was not inconsistent with corresponding information provided by the other two chatbots (i.e., the information by Gemini and Web-search was either consistent with Perplexity or missing), we used the information provided by Perplexity. We tolerated a difference of two years from doctoral graduation and discrepancies in the specification of doctoral program specialization if they were classified in the same way across the chatbots. Second, if the information on a given referee provided by Perplexity was missing or incomplete and the information by Gemini and Web-search was consistent between the two chatbots, we used that information. Third, we copy-pasted the combined prompt, including the professor’s name, to ChatGPT 4o GUI. If ChatGPT returned information consistent with at least one of the three chatbots, we used the information. Fourth, in all other cases, we collected the information manually. To do so, we first tried identifying a referee’s CV in PDF using the following combination of Google search keywords: “’FULL_NAME’ professor finance CV PDF.” If we could not find the CV, we tried to retrieve the information from the reviewer’s LinkedIn profile using the following combination of Google search keywords: “FULL_NAME’ professor finance LinkedIn.” In some cases, we performed unstructured searches and retrieved the information from profiles on personal websites in magazine articles, or similar. We left the data missing if neither of the above approaches worked.

In the second round of data collection, we found that Google Scholar’s dedicated API generally provides accurate responses regarding the information available on Google Scholar profile pages. However, for more complex prompts, the automated loop often returns an error message after processing data for approximately 10 reviewers, even when the information is readily accessible. When the Python code is rerun for the next batch of referees, the API successfully retrieves the data. We attribute this issue primarily to restrictions imposed by Google Scholar (Alphabet) on large-scale data collection. To address this, we balanced the number of variables retrieved with the batch size, adjusting the number of referees processed simultaneously.

For the second round of data collection, we collected the data on all referees in our sample using (1) Google Scholar API, (2) Perplexity API, (3) Gemini by Poe API, and (4) Web-search by Poe API. We applied the following rules. First, if the information about a given referee generated by the Google Scholar API was complete, we used it. Second, if the information about a given referee provided by Perplexity and Gemini did not differ by more than 20%, we used the average of the two numbers. Third, if the information on a given referee provided by Perplexity and Web-search did not differ by more than 20%, we used the arithmetic average of the two numbers. Fourth, in all other cases, we collected the information manually from the Google Scholar website. To find it, we used the following combination of Google search keywords: “FULL_NAME’ professor finance Google Scholar.” We left the data missing if neither of the above approaches worked (typically for academics without a Google Scholar profile).

## Abbreviations

CV: Curriculum vitae
GUI: Graphical user interface
JF: Journal of Finance
JFE: Journal of Financial Economics
SAGER: Sex and gender equity in research
SSCI: Social science citation index
